# Humoral and cell-mediated immune responses after a booster dose of HBV vaccine in HIV-infected children, adolescents and young adults

**DOI:** 10.1371/journal.pone.0192638

**Published:** 2018-02-14

**Authors:** Vania Giacomet, Michela Masetti, Pilar Nannini, Federica Forlanini, Mario Clerici, Gian Vincenzo Zuccotti, Daria Trabattoni

**Affiliations:** 1 Department of Pediatrics, University of Milan, L. Sacco Hospital, Milan, Italy; 2 Chair of Immunology, Department of Biomedical and Clinical Sciences “L. Sacco”, University of Milan, Milan, Italy; 3 Department of Pathophysiology and Transplantation, University of Milan, Milan, Italy; 4 Department of Pediatrics, University of Milan, Ospedale dei Bambini V. Buzzi, Milan, Italy; 5 Don C. Gnocchi Foundation ONLUS, IRCCS, Milan, Italy; University of North Carolina at Chapel Hill School of Medicine, UNITED STATES

## Abstract

**Objective:**

HBV vaccine induces protective antibodies only in 23–56% of HIV-infected children. The aim of our study is to evaluate the immunologic effects of a booster dose of HBV vaccine in HIV-infected youth.

**Design:**

53 young HIV-infected patients in whom HBV vaccination did not elicit protective Ab titers were enrolled. All patients were on ART with optimal immunological and viral response.

**Method:**

All patients received a booster dose of HBV vaccine (HBVAXPRO 10 μg i.m.). HBV-specific Ab titer, viral load and CD4+ T cells were measured at baseline (T0), T1, T6 and T12 months. In a subgroup of 16 patients HBV-specific cell mediated immune responses were evaluated at baseline, at T1 and T6.

**Results:**

The booster dose induced seroconversion in 51% of patients at T1, 57% at T6, and49% at T12; seroconversion rate was significantly correlated with CD4+T cells at T0 and to the CD4 nadir. The booster dose induced HBV-specific cell mediated immunity at T6 mainly in Responders (Rs): Effector Memory CD8+T cells, HBV-specific TNFα-, IFNγ-, granzyme secreting CD8+ T cells and IL2-secreting CD4+ T cells were significantly increased in Rs compared to T0. In Non Responders (NRs), HBV-specific IL2-secreting CD4+ T cells, Central and Effector Memory CD8+ T cells were the only parameters modified at T6.

**Conclusions:**

Seroconversion induced by a booster dose of vaccine correlates with the development of T cell immunological memory in HIV-infected patients who did not respond to the standard immunization. Alternate immunization schedules need to be considered in NRs.

## Introduction

Chronic viral hepatitis has become a major source of comorbidity in Human Immunodeficiency Virus (HIV) infected populations, with improved survival due to the success of antiretroviral therapy (ART). A high incidence of both acute and chronic HBV infection is seen in HIV-infected patients, probably because both viruses share the same route of transmission [1]. Notably, HIV complicates the natural course of HBV, resulting in greater levels of HBV viremia, higher rate of HBV reactivation, chronic hepatitis and increased incidence of cirrhosis and liver-related mortality [2,3].

Immune responses to vaccination, seroconversion rates and antibodies titers induced by vaccination are known to be sub-optimal in HIV-infected people, compared with the healthy population [4–7]. Evaluation of HBV vaccine efficacy is based on HBs antibody titers in serum; titers above 10 IU/ml are considered protective as they are associated with protection [8,9]. Nevertheless, it is estimated that between 4% and up to 10% of healthy individuals vaccinated for HBV fail to achieve protection (anti-HBs below 10 IU/ml) as they are considered non-responders. In addition, between 13 to 60% of vaccinated healthy individuals eventually lose their protection as anti-HBs titers tend to decrease over time, a phenomenon known as “waning immunity” [10,11]. The clinical importance of this phenomenon is however unclear as it has been suggested that protection may still be maintained despite declining anti-HBs Ab titers [12].

In the settings of HIV infection, it has been shown that CD4 counts and viral load at the time of vaccination are the main factors affecting the possibility of observing seroconversion [13]. However, response rates to vaccination differs widely between studies, possibly because of wide differences in the immune-virological status of the patients analyzed, the vaccine administration route and the dosing of vaccination [14,15]. Limited data suggest that it is possible to increase HBV-vaccine response rate in healthy and HIV-infected individuals by modifying vaccine dosing regimens, such as doubling the standard antigen dose.

The production of HBV-specific antibodies by B lymphocytes needs the activation of those CD4+ T helper lymphocytes that will elicit B cell proliferation and their differentiation into Ab-secreting plasmacells. The adjuvants included in HBV vaccines, such as alum, stimulate the migration of dendritic cells (DCs) towards the site of injection and, after antigen capture, their homing into the lymph nodes, where antigens will be processed and presented to CD4+ T cells. These CD4+ T cells will trigger antibody production by secreting cytokines, thus establishing a long-lasting immune memory [16,17]. Notably, it is conceivable that a fully effective vaccine would elicit CTL-mediated effector mechanisms as well, as these are essential in clearing HBV infections [18].

Current guidelines recommend a booster dose of HBV vaccine in HIV-infected patients in whom seroconversion is not achieved but there is still controversy about the risk of viral load rebound in HIV-infected patients, especially in pediatric populations [19].

We analyzed the immunological effects of a booster dose of HBV vaccine on HBV-specific humoral and cell-mediate immune responses in HIV-infected young patients.

## Materials and methods

### Study population

The present study is non-randomized, controlled and approved by Ethical Committee of “L. Sacco” Hospital, Milan, Italy. Fifty-three vertically HIV-infected children, adolescents and young adults, aged from 7 to 25 years, of both sexes, were enrolled in the study at the Pediatric Infectious Diseases Unit at Sacco Hospital (Milan, Italy) and received a booster dose of HBVAXPRO^®^ vaccine, administered by intramuscular injection into the deltoid muscle, as approved by current CDC guidelines(19). Inclusion criteria were: non protective anti-HBs titer (<10 IU/ml), negative anti-HBc titer, completed vaccination schedule, on ART (≥3 antiretroviral, ≥2 classes) for at least one year, good compliance to therapy, informed consent collected from parents and/or legal guardians at enrollment, as appropriate. Exclusion criteria were: presence of comorbidities, chronic therapy with immunosuppressants and/or other immunomodulatory drugs in the 6 months prior to study entry, blood and/or blood products transfusions in the 3 months prior to vaccination, poor compliance to ART, protective anti HBs titers (≥10 IU/ml), history of allergic reactions to a previous vaccination or hypersensitivity to a vaccine component, pregnancy and/or lactation, alcohol and/or drug abuse.

HIV-RNA and CD4+ T cells (absolute number and percentage) were assessed at baseline (T0), after 30 ± 7 days (T1), after 6 months ± 7 days (T6) and 12 months ± 7 days (T12) after the booster dose. Viral load was measured with the lower limit of 37 copies/ml (3.0 Quantiplex assay; Bayer Diagnostics). At each time a clinical evaluation was performed. The study was conducted in accordance with the ethical principles outlined in the Declaration of Helsinki and in accordance with national and European guidelines on clinical observational studies.

### Evaluation of antibody responses to HBV vaccine booster dose

For each patient, venous whole blood was collected for the dosage of anti-HBs antibodies in the following times: at baseline (T0), T1, T6 and T12 after the booster dose. The antibodies titers were assayed using the "Abbott system Architect anti-HBs assay", according to the procedures suggested by the manufacturer.

### Immunophenotypic analysis on PBMCs

A complete evaluation of HBV-specific immunological memory concerning the different functional subtypes of HBV-specific circulating T cells was performed in a subgroup of 16 patients, 9 Responders (Rs) and 7 Non-responders (NRs) after the booster dose at T0, T1 and T6.

The immunophenotypic lymphocyte subpopulations were identified by flow cytometry on previously isolated and frozen peripheral blood mononuclear cells (PBMCs). The following CD4+ and CD8+ T cell subsets were analyzed: Naïve (CD4 +, CCR7+, CD45RA+, CD8+, CCR7+, CD45RA+); Central Memory (CD4+, CCR7+, CD45RA-, CD8+, CCR7+, CD45RA-); Effector Memory (CD4+, CCR7-, CD45RA-, CD8+, CCR7-, CD45RA-); Terminally Differentiated (CD4+, CCR7-, CD45RA+, CD8+, CCR7-, CD45RA+). Activated memory (CD45RO+ and CD38+) CD8+ T cells were also assessed. PBMCs were incubated for 15 minutes at room temperature with the following monoclonal antibodies: anti-human CD4-PeCy7 (eBioscience, USA), anti-human CD8-PC5 (Beckman Coulter, California, USA), anti-human CD38-PE (Biolegend, California, USA), anti-human CD45RO-FITC (Beckman Coulter, California, USA), anti-human CD45RA-FITC (Beckman Coulter, California, USA) and anti-human CCR7-PE (R&D Systems, Minneapolis, USA). Then, the cells were washed with phosphate buffered saline (PBS, Euroclone, Milan, Italy) and fixed with paraformaldehyde (PFA, Sigma-Aldrich) 1%. The lymphocyte population was gated on the basis of its forward and side scatter properties, and further gated for CD4 or CD8 expression; at least 20,000 events were acquired within the CD4 or CD8 gate. The samples were acquired using a Gallios flow cytometer, and the data were analysed using Kaluza software (Beckman Coulter, California, USA).

### HBV-specific cytokine production

PBMCs were cultured in RPMI 1640 medium (Euroclone, Milan, Italy) supplemented with 1% L-glutamine (Sigma-Aldrich), 1% Penicillin/Streptomycin (Sigma-Aldrich) and 10% Human AB Serum (Euroclone, Milan, Italy). For intracellular cytokines evaluation, PBMCs were incubated for 18 hours either in absence or in presence of purified recombinant Hepatitis B Surface Antigen (HBsAg, 1 μg/ml), adw subtype (Acris Antibodies, Germany). Anti-CD28 antibody (R&D Systems, Minneapolis, MN, USA) was added during incubation (2 μg/mL) to facilitate co-stimulation. During the last 6 h of stimulation, 1μg/ml of Brefeldin A (Sigma-Aldrich) was added to block protein secretion. After 18 hours, PBMCs were incubated with monoclonal antibodies to detect surface antigens for 15 minutes at room temperature, fixed with PFA 1%, permeabilized with Saponin 0.5% (sigma-Aldrich) and stained with antibodies for detecting intracellular cytokines for 45 minutes on ice. The cells were then fixed with 1% PFA. The following antibodies were used: anti-human CD4-PeCy5 (eBioscience, USA), anti-human CD8-PC5 (Beckman Coulter, California, USA), anti-human IL-2-PE (eBioscience, USA), anti-human Granzyme B-PE (R&D Systems, Minneapolis, USA), anti-human IFNγ-FITC (eBioscience, USA), anti-human CCR7-PE (R&D Systems, Minneapolis, USA) and anti-human TNFα-PE (Biolegend, USA). The lymphocyte population was gated on the basis of its forward and side scatter properties, and further gated for CD4 or CD8 expression; at least 20,000 events were acquired within the CD4 or CD8 gate.. The samples were acquired using a Gallios flow cytometer, and the data were analysed using Kaluza software (Beckman Coulter, California, USA). Background responses from unstimulated cell cultures were not subtracted from HBV-stimulated responses as they were uniformly low.

### Statistical analysis

Descriptive statistics were reported as percentiles. Median and quartiles values were calculated for age, CD4^+^ T cell counts both in absolute number and percentages at each time point. Tobit analysis was used to estimate the relationship between the rate of seroconversion and CD4^+^ T cells, both in absolute number and in percentage, at each time point. All tests were conducted at a significance level of ≥5%. Fractional polynomials were used to evaluate if the CD4^+^ relationship with seroconversion was linear. Patients were defined as Responders (R) based on the HBsAb level >10 IU/ml at T1 after the booster dose. One Way Anova for repeated measures with Tukey post test for multiple comparisons was used to evaluate the distribution of T-cell subsets and functional subtypes of HBV-specific circulating T cells mean values between responders (anti-HBs titer >10 IU/ml at T1) and non responders (anti-HBs titer <10 IU/ml at T1) at T0, T1 and T6. All statistical analysis were performed using Stata version 14.0. (Stata Corp, Collage Station, TX, USA) and GraphPad Prism 5.

## Results

### Study population characteristics

Seven patients were 7–12 years old, 15 between 13 and 18 years old, and 31 older than 18 years old. Seroconversion rate (anti-HBs ≥ 10 IU/mL) was 51% at T1 (27 patients), 57% at T6 (30 patients), and 49% at T12 (26 patients). At each time point, all patients showed undetectable viral load. The number of NRs was higher in older patients, as showed in Table 1.

**Table 1:** Stratification by age of patients with non-protective antibodies titer (HBAb<10 IU/mL, non-responder, NR) for each time of the study (T1, T6, T12).

	HBsAb<10 IU/mL baseline	NR T1 number (%)	NR T6number (%)	NR T12number (%)
7–12 years	7	2 (29%)	1 (14%)	2 (29%)
13–18 years	15	8 (53%)	6 (40%)	8 (53%)
>18 years	31	16 (52%)	16 (52%)	17 (54%)
total	53	26 (49%)	23 (43%)	27 (51%)

**Fig 1:**
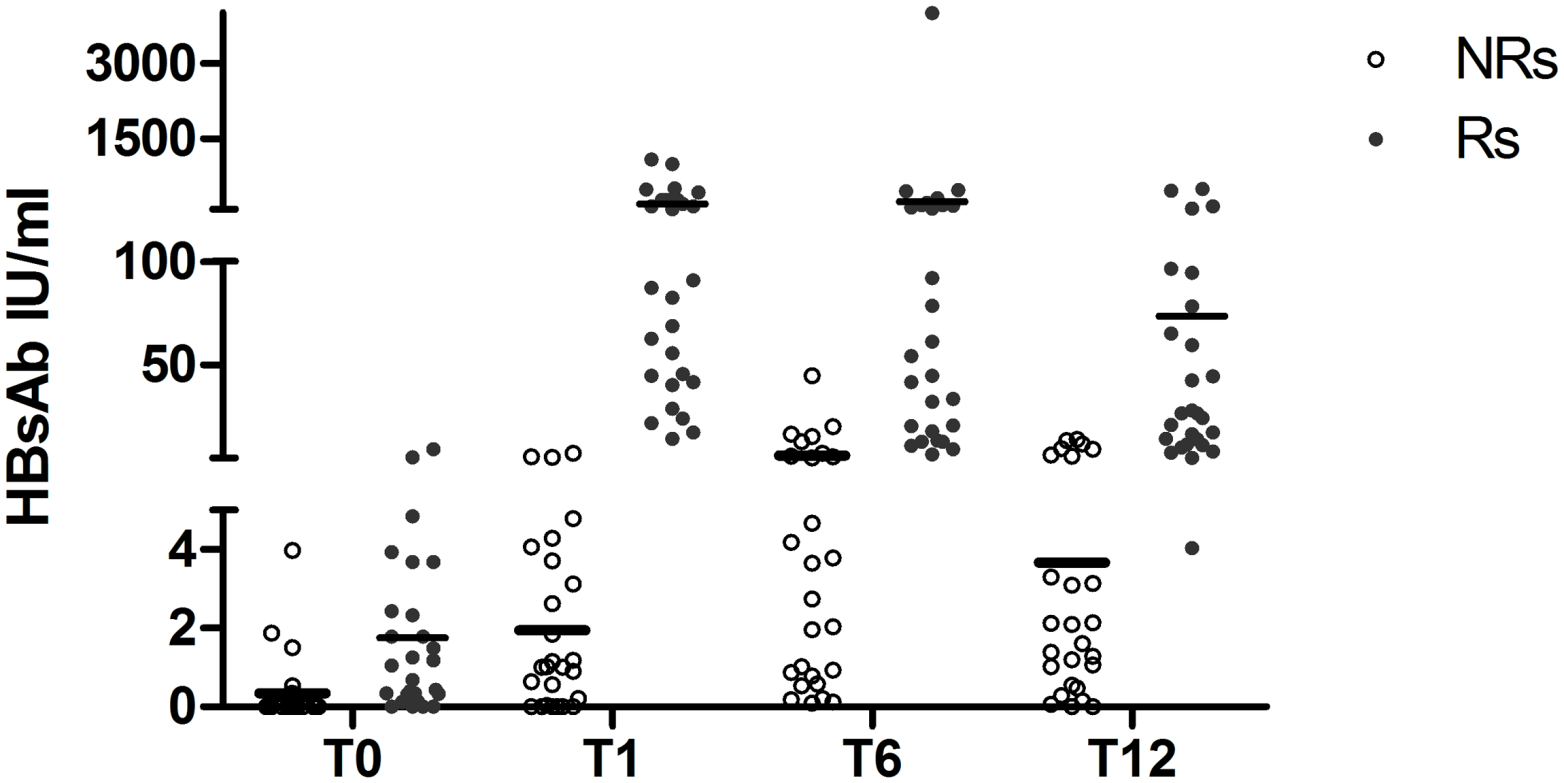
Distribution of HBsAb titers (IU/mL) of Responders (Rs) and Non Responder (NRs) patients at T0, T1, T6 and T12. Horizontal lines indicate means.

Fig 1 shows the mean values of HBs Ab titers of R and NR group at each time point. Median and percentiles values of CD4 at each time point are shown in Table 2.

**Table 2:** Median and percentiles of CD4 count values in absolute number and percentages at T0, T1, T6, T12 and nadir. Tobit analysis showed positive relationship between CD4 nadir (absolute number) and HbsAb seroconversion rate at T1(p<0.01), T6 (p<0.01) and T12 (p<0.05).

	P _25_	P _50_	P _75_
**CD4 T0 absolute number cell/mm**^**3**^ **(%)**	608,5 (28,9%)	713,5 (34,5%)	910,5 (40,32%)
**CD4 T1 absolute number cell/mm**^**3**^ **(%)**	535,25 (29,35%)	750 (34,5%)	923,5 (42,82%)
**CD4 T6 absolute number cell/mm**^**3**^ **(%)**	561,5 (28,2%)	737,5 (34,75%)	878,5 (43,62%)
**CD4 T12 absolute number cell/mm**^**3**^ **(%)**	587 (28,8%)	728 (36,2%)	887 (42,87%)
**Nadir CD4 absolute number cell/mm**^**3**^ **(%)**	180 (11,6%)	306 (17,5%)	397 (26,42%)

Fractional polynomials showed linearity in all relations. Tobit analysis showed the presence of a positive relationship between CD4 nadir and the rate of seroconversion at T1 (p<0.01), T6 (p<0.01) and T12 (p<0.05). A positive and significant relationship was found also between CD4 counts at T0 and the rate of serocoversion at T6 (p<0.05) and T12 (p<0.05).

### Naïve and memory T cells subsets

**Fig 2:**
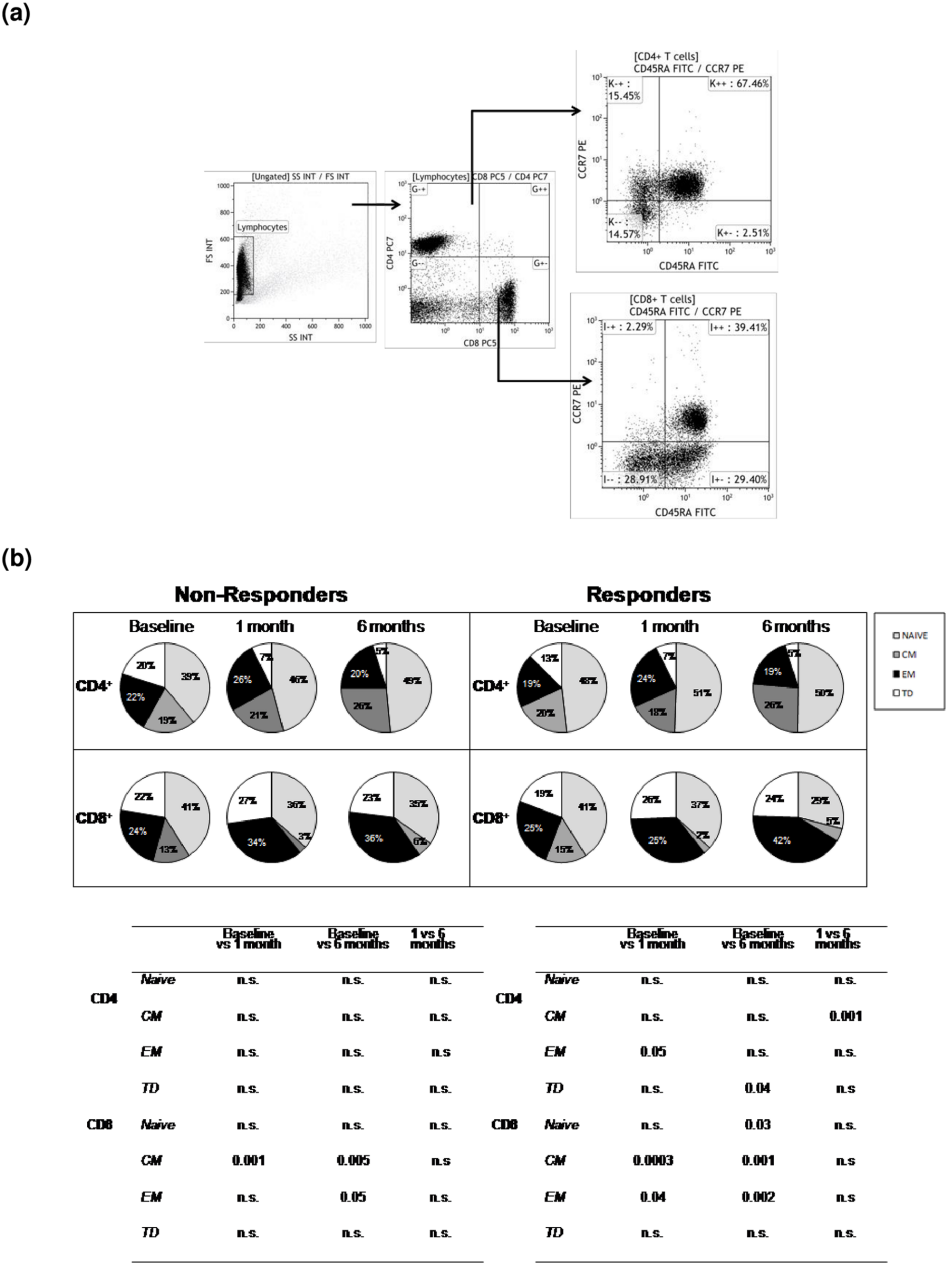
Naïve and memory T cells subsets. (A) Gating strategy for CD4+ and CD8+ T cell subsets analysis (B) Proportion of CD4+ and CD8+ T-cell subsets in Responder and Non Responder individuals. Immunological parameters analyzed in vaccine recipients are shown at baseline and after the booster HBV vaccine dose (T1 and T6). Mean values and statistically significant differences are indicated. NS, not significant. CM, central memory; EM, effector memory; TD, terminally differentiated.

**Fig 3:**
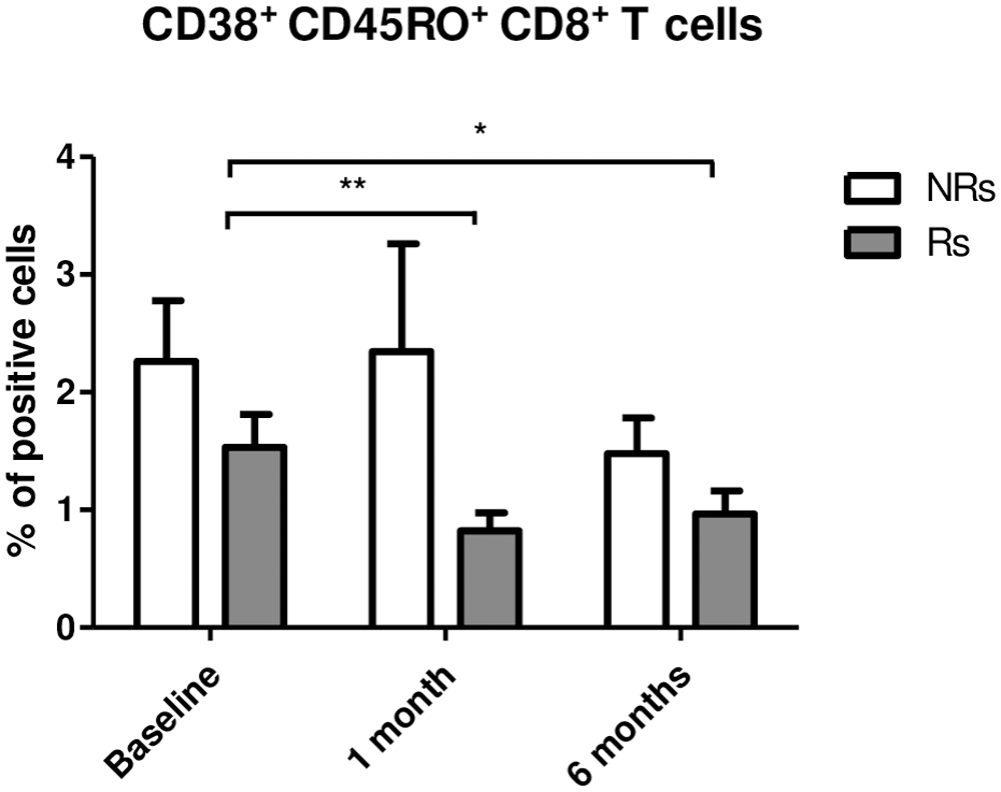
Percentage of CD38+CD45RO+ CD8+T cells. Activated memory CD8+ T cells analyzed in Responder and Non-Responder HIV-infected individuals at Baseline and in response to HBV vaccine booster dose (T1 and T6). * p<0.05, **p<0.01. Mean values and SE are indicated.

CD4+ central memory (CD45RA-, CCR7+) T cells were significantly increased at T6 in Rs only, even though a similar trend could be observed in NRs as well, suggesting the development of a T-cell memory after the vaccine booster dose. Major differences were observed in CD8+ T lymphocytes. Thus, compared to baseline, central memory CD8+ T cells were reduced at T1 and T6 in both Rs and NRs, whereas CD8+ effector memory T cells were significantly increased at T6 in Rs only (Fig 2B). Finally, activated CD38+ CD45RO+ memory CD8+ T cells were significantly reduced at T1 and T6 compared to baseline in responders alone (Fig 3).

### Cell-mediated immune responses (CMI)

**Fig 4:**
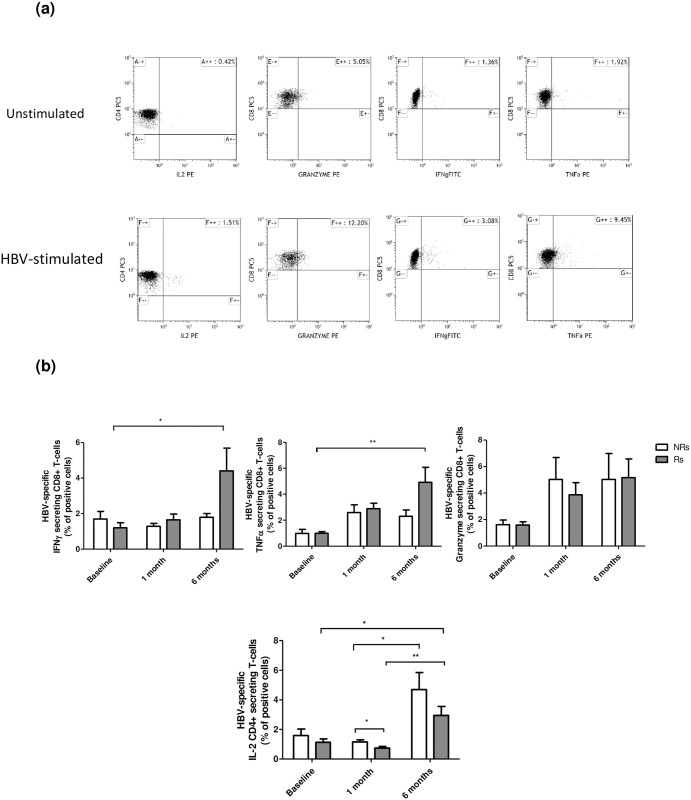
Cell-mediated immune responses. (A) Gating strategy for analysis of CD4+ and CD8+ T cell cytokine secretion without stimulus or after stimulation with HBsAg peptide(B) Percentage of HBV-specific Granzyme-, IFNγ-, and TNFα-secreting CD8+ T-cells and HBV-specific IL-2-secreting CD4+ T-cells analyzed in Responder and Non-Responder HIV-infected individuals at baseline and in response to HBV vaccine booster dose. * p<0.05, **p<0.01.

HBV-specific IFNγ and TNFα-secreting CD8+ T cells were significantly increased at T6 compared to baseline (Fig 4B) in responders alone. These data suggest that the vaccine booster dose in responder patients correlates with the development of an effective immunological memory and with the differentiation of CTL effector mechanisms, which are essential in clearing HBV infections[18]. In contrast with these observations, an increase of HBV-specific IL2-secreting CD4+ T cells at T1 and T6 compared to baseline was detected in both groups (Fig 4B).

## Discussion

Current guidelines recommend a 3-doses HBV vaccine regimen for children and adolescents <19 year-old and it has been demonstrated that this regimen delivers a good response, which is estimated to be around 90% [20–22]. However, immunization response to standard HBV vaccine regimen drops to 28–78% in HIV-infected children and it generally fails to elicit a strong and long-lasting immunity [21]. Studies have shown that re-vaccination or doubling doses of vaccine are able to increase seroconversion rates in HIV-infected children, especially in the settings of well-controlled viremia and immune recovery [23,24].

We decided to give a booster dose of HBV vaccine (HBVAXPRO 10 μg i.m.) to non-responders HIV-infected children after standard vaccination, according to the Italian schedule, in order to evaluate its efficacy in eliciting the production of protective antibodies and CMI responses.

We observed that a booster HBV vaccine dose resulted in a seroconversion rate in 51% of patients at T1, 57% at T6 and 49% at T12 and that it was significantly correlated with CD4+ T lymphocyte counts at T0 and to CD4 nadir, which is in agreement with other reports [reviewed in 22]. Responders patients showed a significant increase in CD8+ EM T cells at T1 and T6 compared to baseline, suggesting that vaccine administration was able to elicit a protective immunological recall response. This observation is in agreement with another recent report in which a more differentiated maturation profile of CD8+ EM T cells was observed in HBV-vaccination responders [25]. Vaccine administration did not seem to increase activation in CD8+ memory T cells and, especially in responders, activation marker CD38 was reduced on memory T cells both at T1 and T6 after vaccine administration. In the subgroup of patients analyzed, responders showed marked HBV-specific cell mediated immune responses, especially at T6 compared to baseline. In particular, we observed increased CD8+ CTL effector functions specifically in Rs with significant increases of TNFα and IFNγ secretion, suggesting that in Rs, T cells effector functions are more potentiated than in NRs after a booster dose of vaccine. In agreement with our data, a recent work analyzing immunological response to Hepatitis B vaccination in treated HIV infection, has shown that IFNγ secretion by CD8+ T cells after Hepatitis B-specific stimulation is significantly increased in responders only [25]. Other reports studying T-cell immunity after HBV vaccination have shown that responders to vaccination display increases in cytokine production, especially Th1 cytokines, whereas non-responders fail to mount a cytokine response when challenged with HBV antigens, which is in agreement with our data [26–29].

Taken together these data suggest that responders patients are characterized by a more responsive antigen-specific adaptive cell-mediated immunity, especially in the CD8+ T cell compartment. In both groups, we observed increased HBV-specific CD4+ IL2-producing T cells at T6 compared to baseline confirming previous observations suggesting that immunological memory provided by HBV-specific T cells may be sufficient for a recall response [30]. In addition, these data suggest that booster doses of HBV vaccine may increment the HBV-specific immune responses, especially in individuals who have experienced waning immunity after primary vaccine series.

In conclusion, in HIV-infected children, adolescents and young adults not responding to the standard HBV immunization protocol or with waning immunity, seroconversion induced by a booster dose of vaccine has been shown to be safe, as no serious side effect was reported by the patients, and to correlate with the development of humoral and cellular immune responses and memory.

Alternate immunization schedules should be designed and implemented for those individuals who do not respond even to a booster dose of vaccine.
